# Pelvic floor and perineal muscles: a dynamic coordination between skeletal and smooth muscles on pelvic floor stabilization

**DOI:** 10.1007/s12565-023-00717-7

**Published:** 2023-03-24

**Authors:** Satoru Muro, Keiichi Akita

**Affiliations:** Department of Clinical Anatomy, Tokyo Medical and Dental University, 1-5-45 Yushima, Bunkyo-Ku, Tokyo, 113-8510 Japan

**Keywords:** Pelvic floor muscles, Perineal muscles, Perineal body, Skeletal muscle, Smooth muscle

## Abstract

The purpose of this review is to present our researches on the pelvic outlet muscles, including the pelvic floor and perineal muscles, which are responsible for urinary function, defecation, sexual function, and core stability, and to discuss the insights into the mechanism of pelvic floor stabilization based on the findings. Our studies are conducted using a combination of macroscopic examination, immunohistological analysis, 3D reconstruction, and imaging. Unlike most previous reports, this article describes not only on skeletal muscle but also on smooth muscle structures in the pelvic floor and perineum to encourage new understanding. The skeletal muscles of the pelvic outlet are continuous, which means that they share muscle bundles. They form three muscle slings that pass anterior and posterior to the anal canal, thus serving as the foundation of pelvic floor support. The smooth muscle of the pelvic outlet, in addition to forming the walls of the viscera, also extends in three dimensions. This continuous smooth muscle occupies the central region of the pelvic floor and perineum, thus revising the conventional understanding of the perineal body. At the interface between the levator ani and pelvic viscera, smooth muscle forms characteristic structures that transfer the lifting power of the levator ani to the pelvic viscera. The findings suggest new concepts of pelvic floor stabilization mechanisms, such as dynamic coordination between skeletal and smooth muscles. These two types of muscles possibly coordinate the direction and force of muscle contraction with each other.

## Introduction

The pelvic floor is a structure unique to humans formed by upright bipedal walking (Smith 1923; Thompson 1899). In quadrupeds, the weight of the abdominal viscera rests on the abdominal wall; in humans, it is directed toward the pelvic outlet. This necessitates that the human pelvis must have a mechanism to resist gravity while maintaining function as an exit for reproduction and excretion. The pelvic floor is the bearer of this mechanism; that is, the structure that supports the abdominal and pelvic organs against gravity.

The pelvic floor is responsible for lower urinary tract function (storing and eliminating urine), defecation (eliminating feces from the digestive tract through the anus), and sexual function (erectile function and ejaculation in men, and sexual sensation and arousal in women) (Bharucha 2006; Corton 2009; Fritsch et al. 2004; Standring and Gray 2015). Therefore, when the pelvic floor support mechanism is weakened or injured, it manifests as pelvic organ prolapse, dysuria, defecation, and sexual dysfunction (Bø 2004; Lawson and Sacks 2018; Quaghebeur et al. 2021). Factors that reportedly contribute to such pelvic floor fragility include multiple births, aging, pregnancy, obesity, menopause, connective tissue disorders, smoking, chronic obstructive pulmonary disease, and chronically elevated intra-abdominal pressure (Ashton-Miller and DeLancey 2007; DeLancey et al. 2003; García Del Salto et al. 2014). The pelvic floor muscles are also attracting attention as a component of core stability because core stability training has been reported to be useful in preventing injury and improving performance in sports (Huxel Bliven and Anderson 2013; Kibler et al. 2006). The pelvic floor muscles, in conjunction with the diaphragm and transversus abdominis, increase intra-abdominal pressure and provide stability to the lumber spine (Akuthota and Nadler 2004). Thus, the pelvic floor is involved in a wide variety of functions and pathologies, including urological, gastrointestinal, obstetric, gynecological, and locomotor diseases. Knowledge of pelvic floor anatomy is critical for the prevention and treatment of pelvic floor dysfunction, quality of life, and overall healthcare.

The pelvic floor is composed of muscles and ligaments; this review focused on these muscles. The muscles are histologically classified as skeletal and smooth. The muscles that comprise the pelvic floor are primarily skeletal, which are called “pelvic outlet muscles.” Based on the findings in mammals, Eggeling (1933) defined the muscles of the pelvic outlet (Die Muskeln des Beckenausgangen) as skeletal muscles, which are closely associated with the distal ends of the urogenital sinus and rectum, forming the closure of the caudal region of the pelvic cavity (Akita 1997; Akita et al. 1995; Eggeling 1933). The pelvic outlet muscles consist of the pelvic floor muscles (pelvic diaphragm) and perineal muscles. The pelvic floor muscles include the levator ani (LA) and coccygeus, and the perineal muscles consist of the bulbospongiosus (Bs), ischiocavernosus (Ic), superficial transverse perineal muscle (STP), and external anal sphincter (EAS) (Standring and Gray 2015; Wei and De Lancey 2004). Comparative anatomical and embryological studies have shown that the pelvic outlet muscles are derived from the ventral muscles of the hind limbs (Akita 1992a, b, 1997; Akita and Yamamoto 1995; Akita et al. 1992a, b, 1994a, b, 1995; Valasek et al. 2005). The pelvic floor muscles delineate the lower limit of the true pelvis and separates the pelvic cavity above from the perineal region below. The LA is essential for supporting the abdominal and pelvic organs, while the perineal muscles play a role in controlling the passage of the urethra, vagina, and anal canal (Cunningham and Romanes 1981). Previously, when we speak of the pelvic outlet muscles, we refer to the skeletal muscles of the pelvic outlet, the structure of which has conventionally been the subject of analysis.

However, we focused not only on skeletal muscle, but also on smooth muscle. Focusing on the smooth muscles reveals a new understanding of the structure of the pelvic floor. We have reported several studies of both the skeletal and smooth muscles that comprise the pelvic floor, providing a new structural understanding of the pelvic floor. These our studies are anatomical studies performed using a combination of macroscopic examination, immunohistological analysis, 3D reconstruction, and imaging (Muro and Akita 2023). The purpose of this review is to present our researches on the pelvic outlet muscles (pelvic floor and perineal muscles) and discuss the insights into the mechanism of pelvic floor stabilization based on the findings. What is derived from this is a new concept of the pelvic floor support mechanism: dynamic coordination between smooth and skeletal muscles.

## Skeletal muscles of pelvic outlet

### Continuity of skeletal muscles

The anatomy of the skeletal muscles of the pelvic outlet is difficult to understand. It seems that many people who deal with the anatomy of the pelvic floor have assumed a tendinous node called the “perineal body” at the center of the pelvic floor to aid their understanding. Therefore, the perineal body is believed to be a major point of attachment for pelvic floor and perineal muscles (Corton 2009; Oh and Kark 1973; Wu et al. 2015; Zhai et al. 2011). However, this seems to have spread misconceptions about the arrangement of the pelvic outlet muscles, especially the perineal muscles.

**Fig. 1:**
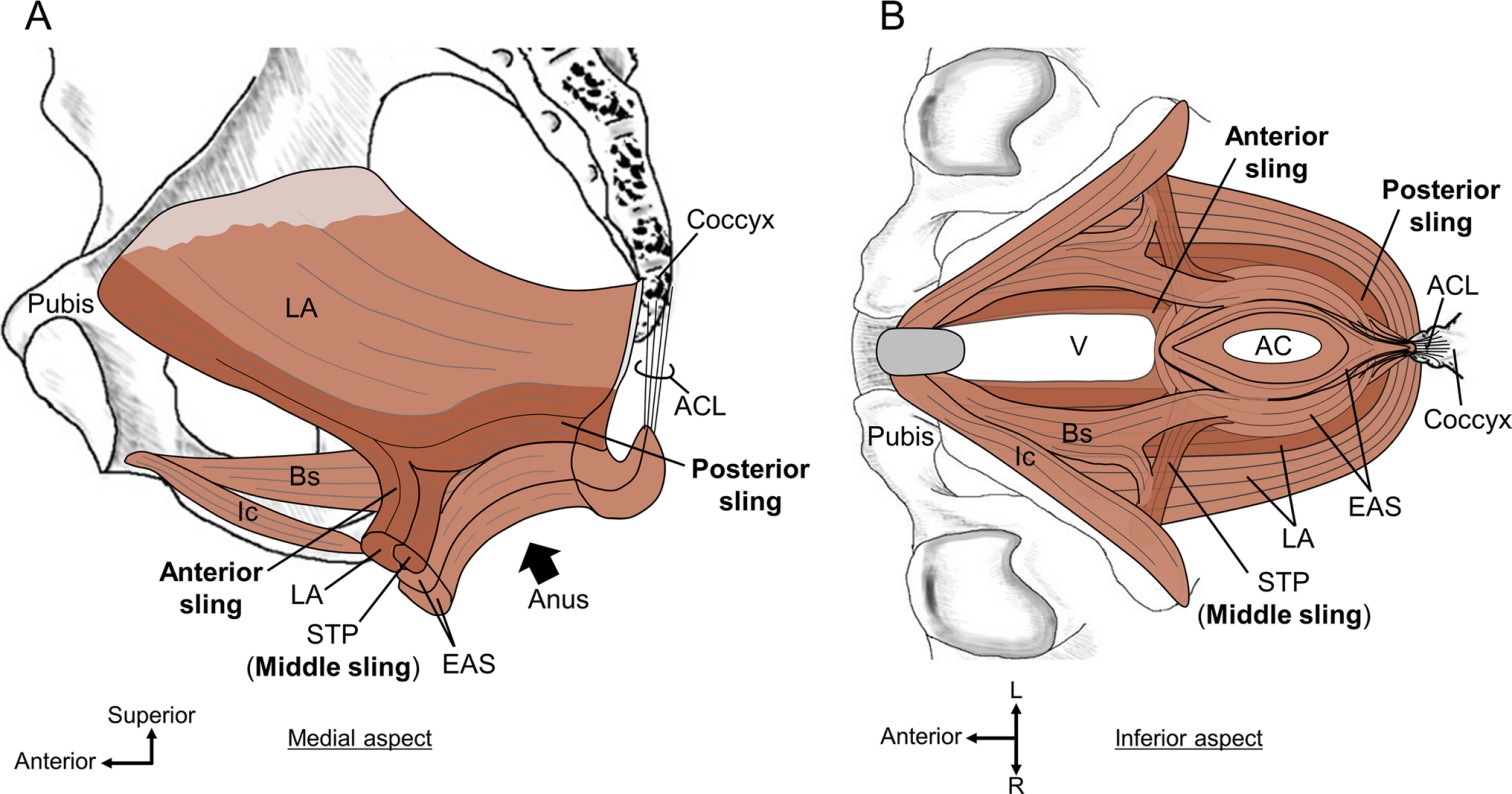
Skeletal muscles of the pelvic outlet in women. The skeletal muscles of the pelvic outlet share muscle bundles and are continuous, forming a continuous skeletal muscle sheet. **A** The pelvic outlet muscles in women from the medial aspect. The LA surrounds the anal canal both anteriorly and posteriorly; the anterior sling and posterior sling of the LA go around the anterior and posterior to the anal canal, respectively. Bs does not run toward the midline but goes laterally. The STP crosses the midline and continues as the same muscle to the contralateral side, forming a component of the anal canal anterior wall. **B** The inferior aspect of the pelvic outlet muscles in women. Bs adjoins the lateral surface of the EAS to form a lateral connection between Bs and EAS. The anterior sling of LA, muscle bundle of STP, and posterior sling of LA cross the midline and then merge with the contralateral side to form three muscle slings that pass anterior and posterior to the anal canal: anterior, middle, and posterior. *AC* anal canal, *ACL* anococcygeal ligament, *Bs* bulbospongiosus, *EAS* external anal sphincter, *Ic* ischiocavernosus, *LA* levator ani, *STP* superficial transverse perineal muscle, *V* vagina. (Modified from: Baramee et al. 2020)

**Fig. 2:**
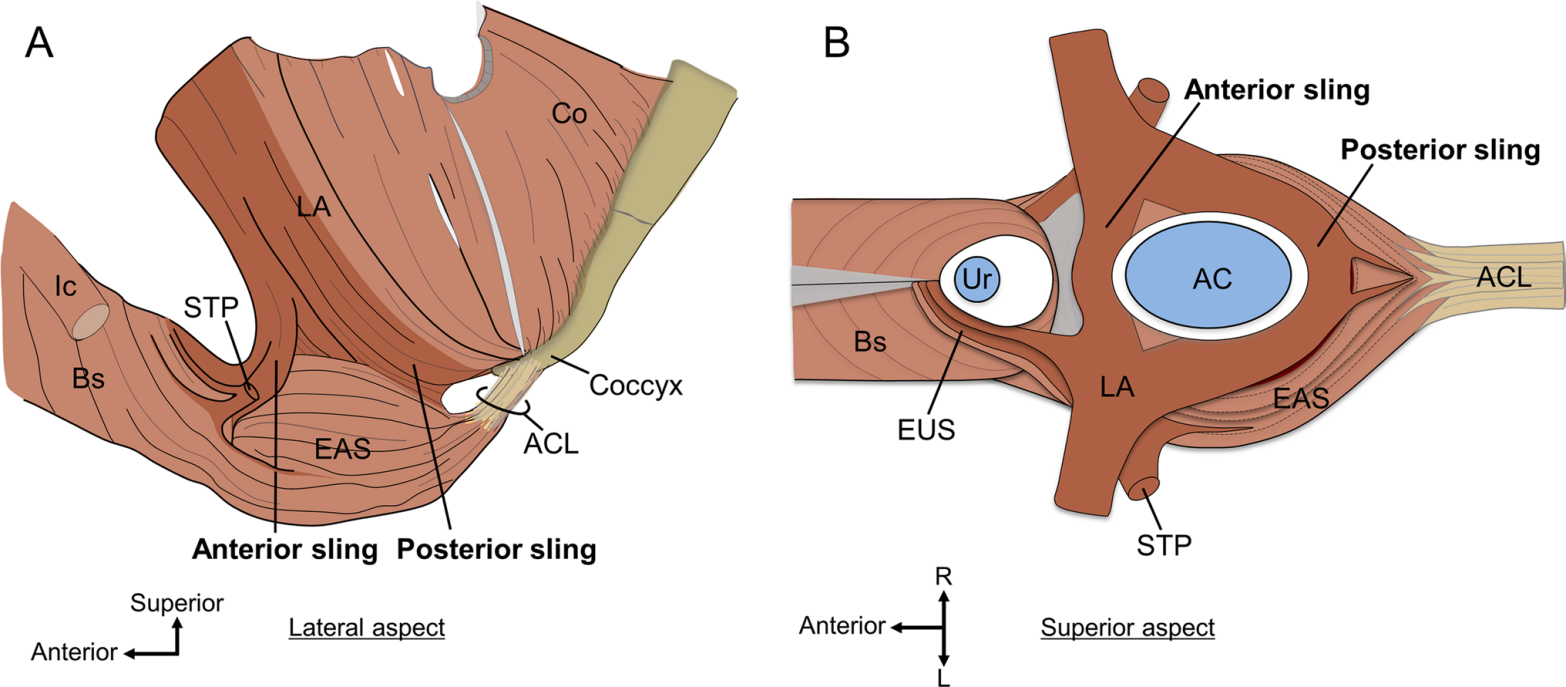
Skeletal muscles of the pelvic outlet in men. The skeletal muscles of the pelvic outlet share muscle bundles and are continuous, forming a continuous skeletal muscle sheet. **A** The pelvic outlet muscles in men from the lateral aspect. The LA surrounds the anal canal both anteriorly and posteriorly; the anterior sling and posterior sling of the LA go around the anterior and posterior to the anal canal, respectively. Bs connected inferolaterally to the EAS. The STP has muscle bundles that connect anteriorly to the Bs and posteriorly to the EAS. **B** The superior aspect of the pelvic outlet muscles in men. The anterior and posterior slings of LA cross the midline and then merge with the contralateral side to pass anterior and posterior to the anal canal. Some fibers of the LA, STP, and EAS extend anterosuperiorly, enclosing the lateral and anterior sides of the urethra, forming EUS. *AC* anal canal, *ACL* anococcygeal ligament, *Bs* bulbospongiosus, *EAS* external anal sphincter, *Ic* ischiocavernosus, *LA* levator ani, *STP* superficial transverse perineal muscle, *Ur* urethra. (Modified from: Suriyut et al. 2020)

The most distinctive feature of the skeletal muscles of the pelvic outlet is that they share muscle bundles and are continuous (Figs. 1 and 2). It is well known that LA and EAS are not separated but are continuous with each other (Ayoub 1979b; Baramee et al. 2020; Courtney 1950; Suriyut et al. 2020; Tsukada et al. 2016; Uchimoto et al. 2007; Williams 1995). Additionally, many tiny connecting muscle bundles exist among the skeletal muscles, including the LA, EAS, Bs, Ic, STP, and external urethral sphincter (EUS) (Baramee et al. 2020; Henle 1866; Muro et al. 2021b; Peikert et al. 2015; Plochocki et al. 2016; Suriyut et al. 2020). Based on these findings, it should be recognized that the LA and perineal muscles form a continuous skeletal muscle sheet and act as a complex rather than independently. This continuity of skeletal muscles calls into question the conventional dominant concepts of “the skeletal muscles of the pelvic outlet as independent muscles” and “the attachment as the tendinous node called the perineal body.”

We present three important findings that lead to the renovation of the concept that captures the basic structure of the pelvic floor and perineal muscles:

1. “Anterior sling” of the LA;
2. Bs in women does not run toward the center of the perineum, it adjoins the external surface of EAS; and
3. STP in the median merges with the contralateral STP to form the “middle sling.”

These are discussed in detail below.

### Levator ani

The LA, the largest pelvic floor muscle, contains various muscle bundles and has a complex composition. Among them, the following muscle bundles are important when considering pelvic floor support: (1) muscle bundles that surround the anal canal anteriorly and posteriorly; and (2) muscle bundles that attach to the pelvic viscera. Additionally, some muscle bundles attach to the anococcygeal raphe, sacrum, and coccyx; these muscle bundles also are important as pressure septum in the pelvic outlet.

It is widely known that some muscle bundles of the LA form a sling that goes around the posterior to the anal canal, which is generally called “puborectalis” (Fritsch et al. 2007; Holl 1897; Standring and Gray 2015; Stoker 2009; Thompson 1899; Wu et al. 2015). It was recently reported that there is also an “anterior sling” that goes around the anterior to the anal canal (Figs. 1 and 2) (Baramee et al. 2020; Suriyut et al. 2020). The LA surrounds the anal canal both anteriorly and posteriorly. Anterior and posterior slings are found in both men and women.

The portion of the muscle bundles of the LA that attach to the pelvic viscera has long been recognized; however, it is often named the “pubococcygeus” (Kearney et al. 2004). This term is inherited from the term used in animals and does not match the origin or insertion of muscle bundles in humans (Smith 1923). Therefore, Kearney et al. (2004). supported the term “pubovisceralis,” as described by Lawson, instead of the pubococcygeus (Kearney et al. 2004; Lawson 1974). In light of the current dissociation between terms and structures and their confusion, this proposal is worthy of consideration. The form of the attachment to the pelvic viscera is described in detail below.

The more posterior muscle bundles of the LA attach to the connective tissue that is called as the anococcygeal raphe when the bilateral LA meets the posterior median plane (Gordon and Nivatvongs 2007; Gray et al. 2008). Shafik named this connective tissue “anococcygeal raphe” (Shafik 1975, 1979, 1999). It has a collagenous structure in the same plane as the LA. “Coccygeal muscular raphe” described by Courtney (1948, 1949), “levator raphe” described by Oh and Kark (1972), “levator plate” described by Strohbehn (1998), and “dorsal layer of the anococcygeal ligament” described by Kinugasa et al. (2011, 2012) represent the same structure (Courtney 1948, 1949; Kinugasa et al. 2011, 2012; Oh and Kark 1972; Strohbehn 1998). The terminology for the structures located between the anal canal and coccyx is currently not unified, however they can be organized in the stratigraphic relationship with the LA (Table 1).

**Table 1:** Names of the structures between the anal canal and coccyx (Modified from: Muro et al. 2014)

Authors (Year)	Position to the levator ani		
	Ventral side	Same plane	Dorsal side
Toldt (1903)			Anococcygeal ligament (Lig. anococcygeum)
Courtney (1948, 1949)	Iliorectococcygeus muscle	Coccygeal muscular raphe	The decussating fibers of the deep portion of the external anal sphincter as they insert into the skin
Oh and Kark (1972)		Levator raphe	Anococcygeal raphe
Shafik (1975, 1979, 1999)	Hiatal ligament	Anococcygeal raphe	The posterior part of Intermediate loop
Ayoub (1979a)			Anococcygeal ligament
Strohbehn (1998)		Levator plate	
Kinugasa et al. (2011, 2012)	Ventral layer of the anococcygeal ligament	Dorsal layer of the anococcygeal ligament	
Muro et al. (2014)	Hiatal ligament	Raphe of iliococcygeus muscle and pubococcygeus muscle	Anococcygeal ligament

The names of the structures between anal canal and coccyx are listed on the basis of the relative position to the levator ani; same plane, ventral side or dorsal side to the levator ani

A recent topic of interest regarding LA is its relationship to the obturator internus, a muscle of the hip joint. The LA was found to be in broad planar contact with the obturator internus and has several muscle layers attached to the obturator fascia (Muro et al. 2022). Based on these anatomical findings, we assume a functional relationship in which the dynamic movement of the obturator internus cooperates with the LA through the obturator fascia, providing the foundation for the function of the LA. This suggests that balanced and proper movements of the obturator internus contribute to the function of the LA, that is, pelvic floor support. Various aspects of the functional relationship between the LA and obturator internus have been suggested, including reports that hip rehabilitation contributed to the strengthening of the pelvic floor muscles and the improvement of stress urinary incontinence (Jordre and Schweinle 2014; Tuttle et al. 2016), reports that hip function with total hip arthroplasty improved urinary incontinence (Baba et al. 2014; Martines et al. 2022; Okumura et al. 2017; Tamaki et al. 2014), and a report that electrophysiological stimulation of the fascia of the obturator internus resulted in LA contraction (Chin et al. 2021).

### Bulbospongiosus and superficial transverse perineal muscle

The Bs and STP are perineal muscles located in the perineal triangle. Bs cover the bulb of the penis and bulb of the vestibule, whereas STP attaches to the ischial tuberosity and runs transversely. These are small muscles; however, the new findings are significant and call for a shift in our understanding of the basic structure of the pelvic outlet muscles. Previously, Bs and STP have been described as being attached to the perineal body (Standring and Gray 2015). Of course, this assumes the existence of a tendinous node called the perineal body. However, many recent reports have contradicted this finding.

Bs in women originate from the corpus cavernosum and body of the clitoris and run posteriorly on the lateral side of the vagina. Several histological and MRI studies have reported that Bs in women do not attach to the perineal body but pass through it (Arakawa et al. 2010; Mittal et al. 2014; Shafik et al. 2005, 2007). Another study using high-resolution three-dimensional endovaginal ultrasound also pointed out that Bs in women do not move toward the midline (Santoro et al. 2016). Recent detailed anatomical studies have shown that Bs in women do not move toward the midline but attach to the lateral surface of the EAS; that is, there is a lateral connection between the Bs and EAS (Fig. 1) (Baramee et al. 2020; Plochocki et al. 2016). This finding is consistent with the results of 3D reconstruction studies based on MRI and serial human body sections (Larson et al. 2010; Wu et al. 2015). Although Bs and EAS in men are inferolaterally connected, they differ from those in women in that Bs are connected to the contralateral muscle bundle at the median line (Fig. 2) (Muro et al. 2021b; Suriyut et al. 2020). In men, Bs is closed in the midline; therefore, a connection is observed in the inferior midline. It is reasonable that the shape of Bs differs between women, whose urethra and vagina open into the vestibule and the bulb of the vestibule is divided into right and left, and men, whose long urethra is covered by corpus cavernosum tissue, and the single bulb of the penis is located in the midline.

The dominant perception of STP is that it originates from the ischial tuberosity, moves medially, and attaches to the perineal body in the midline in both men and women (Standring and Gray 2015). However, it has long been pointed out that the STP does not end in the midline; however, may cross the midline (Oh and Kark 1973; Shafik et al. 2005). Recent detailed anatomical studies have shown that the STP in women crosses the midline and continues as the same muscle on the contralateral side (Fig. 1) (Baramee et al. 2020). This finding is consistent with the results of 3D reconstruction studies based on MRI and serial human body sections (Larson et al. 2010; Wu et al. 2015). The STP is not a muscle that ends in the midline but rather a muscle that crosses the midline and connects the bilateral ischial tuberosities while passing between the division of the anterior and posterior slings and forming a component of the anterior wall of the anal canal. In addition to the anterior and posterior slings of the LA, an interpretation that the STP is considered a middle sling has been proposed (Baramee et al. 2020). In addition, the muscle bundles of the STP are very diverse, with some muscle bundles adjoining the EAS and others adjoining the Bs (Figs. 1 and 2).

Thus, neither the Bs nor STP attaches to the perineal body. What about the center of the pelvis? Does a perineal body exist? What is a perineal body? These questions are discussed below. Undoubtedly, the morphology of small muscles (Bs and STP) raises questions about the anatomical notion of the perineal body, which has long been considered the cornerstone of the pelvic floor.

### Three-muscle sling theory

If we cannot assume that there is a perineal body or a tendinous attachment point (origin/insertion) in the center of the pelvic floor, the only fixed structures that could be attached to the pelvic floor and perineal muscles are the coxal bone (pubis, ischium, and ilium); sacrum; and coccyx. Many muscle bundles that cross the midline merge with the contralateral side to form a sling structure. Based on the findings of the LA and perineal muscles described above, we can recognize three muscle slings that pass anterior and posterior to the anal canal (Figs. 1 and 2) (Baramee et al. 2020; Suriyut et al. 2020).

- Anterior sling: part of the muscle bundle of the LA. It arises from the pubis, goes inferoposteriorly, crosses the midline anteriorly to the anal canal (posteriorly to the vagina), and joins the contralateral muscular bundle.
- Middle sling: muscle bundle of the STP. It arises from the ischial tuberosity, moves medially, crosses the midline anteriorly to the anal canal (posteriorly to the vagina), and joins the contralateral muscular bundle.
- Posterior sling: part of the muscle bundle of the LA. It arises from the pubis, goes inferoposteriorly, lateral to the rectum, crosses the midline posteriorly to the anal canal, and joins the contralateral muscular bundle.

Appropriate functioning of these three muscle slings is important for pelvic floor support. It is particularly noteworthy that the anterior sling and middle sling are components of the skeletal muscle of the anterior wall of the anal canal, as Baramee et al. (2020) have shown (Baramee et al. 2020). The anterior wall of the skeletal muscle of the anal canal is not solely composed of the EAS. The anterior sling of the LA and middle sling of the STP also constitute the anterior wall of the anal canal. These are part of the wall of the anal canal and slings that suspend the anal canal toward the pelvic bones. This “three muscle slings theory,” based on anatomical findings, could serve as a foundational model for both pelvic floor support mechanisms and defecation function.

## Smooth muscles of pelvic outlet

### Central region of pelvic floor and perineum

The perineal muscles and LA are generally considered to be attached to the “perineal body,” which is described as a mass of fibromuscular or dense connective tissue in the center of the pelvic floor and perineum (Aigner et al. 2004; Corton 2009; Muhleman et al. 2017; Oh and Kark 1973; Plochocki et al. 2016; Shafik et al. 2007; Standring and Gray 2015; Stoker 2009; Woodman and Graney 2002; Wu et al. 2015; Zhai et al. 2011). The perineal body had been generally interpreted as an independent tendinous node (Corton 2009; Wu et al. 2015). However, several researchers offered a different view that the term perineal body should be used as a term to indicate a region rather than a specific structure (Larson et al. 2010; Muro et al. 2018). That is, the “perineal body” should be defined as the region between the rectum, anal canal, and urogenital organs. Larson et al. (2010) explained this definition by using the word “shoulder” as an example (Larson et al. 2010). The “perineal body” refers to the region between the rectum, anal canal, and urogenital organs, the same as the “shoulder” refers to the region between the brachium and trunk (and cervix). If the term “perineal body” was limited to describing a region, confusion could be prevented. Therefore, we suggest using the term “perineal body” to refer to a region rather than a structure.

Additionally, what kind of structures are in the region of the perineal body is smooth muscle. Therefore, the central region of the pelvic floor and perineum is occupied by smooth muscle (Muro et al. 2018, 2019, 2021b; Nakajima et al. 2017). Its morphological characteristics are as follows: (1) smooth muscles are continuous with the walls of the pelvic viscera; (2) they do not form independent muscles but extend continuously; (3) they spread out to fill the space between the skeletal muscles; (4) they have dense and sparse areas in a continuous expanse; and (5) they provide nerve pathways.

Smooth muscles are generally responsible for forming the walls of the viscera and secreting glands. However, in the pelvic outlet region, smooth muscles not only form the walls of the viscera but also extend and spread out in three dimensions (Kato et al. 2020; Kraima et al. 2016; Muro et al. 2018, 2019, 2020, 2021b; Nakajima et al. 2017; Nyangoh Timoh et al. 2020; Okada et al. 2019; Uchimoto et al. 2007; Zhai et al. 2011). The extension from the visceral wall to the surroundings is particularly marked in the region anterior to the rectum and anal canal (Kraima et al. 2016; Muro et al. 2018, 2019, 2020, 2021b; Nakajima et al. 2017). Owing to this extended nature, the smooth muscle structure of the pelvic outlet does not form independent muscles but extends continuously. Individually named smooth muscle structures to intersect and are continuous with each other to form a series of smooth muscle structures. These smooth muscles spread to fill the spaces between two viscera, the viscera and skeletal muscles, and between two skeletal muscles. This results in a complementary arrangement of the skeletal and smooth muscles, as described below. Additionally, the smooth muscle of the pelvic outlet is continuous, but not uniform. There are dense and sparse areas within a continuous structure (Muro et al. 2019, 2020, 2021b). Relatively sparse areas provide pathways for nerves and blood vessels to the pelvic viscera (He et al. 2022; Muro et al. 2019; Nyangoh Timoh et al. 2020). Continuous smooth muscle in the central region of the pelvic floor and perineum is the third player in pelvic floor support, which has conventionally been discussed mainly in terms of skeletal muscles and ligaments. In other words, smooth muscles may also contribute to pelvic floor support.

### Three-dimensional extent of smooth muscle

The smooth muscle that extends outward from the walls of the pelvic viscera spreads three-dimensionally and locally, creating characteristic relationships with the surrounding structures. The three representative elements of smooth muscle in the central region of the pelvic floor and perineum and other extensions to the surroundings are described below (Table 2). It should be noted that each structural element is continuous, with fibers intersecting each other. Although it is possible to artificially define boundaries, there are no morphologically distinct boundaries. They should be understood as the structural elements of a series of smooth muscles.

**Table 2:** Structural elements of the series of continuous smooth muscles of the pelvic outlet (Modified from: Muro et al. 2021b)

Term	Location	Attachment (Origin and insertion)	Fiber direction	Fiber density (Relative to the longitudinal muscle of the rectum)
Rectourethralis (Ru) [Rectovaginalis (Rv)]	The median region between the rectum and urethra [vagina]	– The dorsal portion connects to the longitudinal and circular muscles of the rectum – The ventral portion merges with the smooth muscle of the internal urethral sphincter [the lower posterior vaginal wall]	Dorsoventral	Sparse
Deep transverse perineal muscle (DTP)	Deep perineal pouch	– The lateral portion attaches to the bilateral ischiopubic rami – The medial portion crosses transversely between the rectum and urethra [vagina], and connects to the longitudinal muscle of the rectum	Transverse	Dense
Anterior bundle of the longitudinal muscle (AB)	The median region between the external anal sphincter and bulbospongiosus [skin of the perineum]	– The upper portion merges with the longitudinal muscle of the rectum – The lower portion attaches to the skeletal muscle bundles of the perineal muscles [extends subcutaneously in perineum]	Craniocaudal	Dense
Hiatal ligament (HL)	Between the pelvic viscera (urethra, vagina, rectum, and anal canal) and levator ani	– The medial portion merges with the smooth muscle of the vaginal wall, rectourethralis, and the longitudinal muscle of the rectum – The lateral portion contacts the internal surface of the levator ani	Superolateral	Sparse

#### Rectourethralis and rectovaginalis

**Fig. 3:**
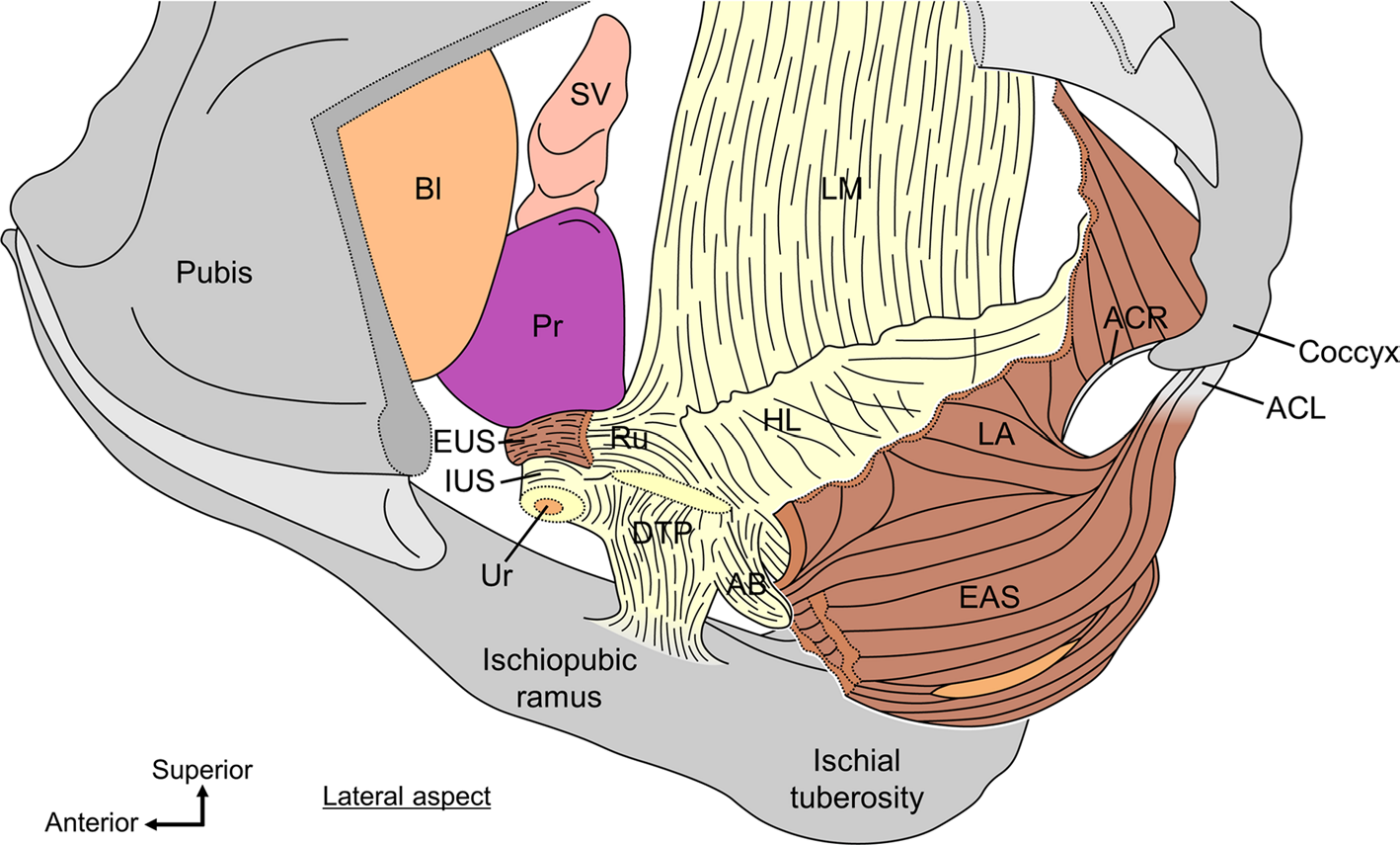
The continuous smooth muscle in the central region of the pelvic outlet in men. The IPR and IT on the left side have been removed, along with the distal urethra and the LA and DTP on the left. Continuous smooth muscle is composed of several structural elements. Ru is a wedge-shaped structure located between the rectum and urethra and consists of smooth muscle fibers that extend from the LM of the rectum toward the smooth muscle of the urethra. The DTP is a plate-like structure located between the rectum and urethra, caudal to the Ru. It crosses the midline, spreads bilaterally, and reaches the IPR, consisting of smooth muscle fibers that extend from the LM of the rectum and anal canal. AB is a columnar structure located in the median region anterior to the EAS, consisting of smooth muscle fibers that extend from the LM of the rectum and anal canal. The HL is the amorphous tissue located between the pelvic viscera (urethra, vagina, rectum, and anal canal) and LA, consisting of smooth muscle fibers that extend from the vaginal wall, Ru, and LM of the rectum. The ACL connects the anal canal to the coccyx. *AB* anterior bundle of LM, *ACL* anococcygeal ligament, *ACR* anococcygeal raphe, *Bl* bladder, *DTP* deep transverse perineal muscle, *EAS* external anal sphincter, *EUS* external urethral sphincter, *HL* hiatal ligament, *IUS* internal urethral sphincter, *LA* levator ani, *LM* longitudinal muscle, *Pr* prostate, *Ru* rectourethralis, *SV* seminal vesicle, *Ur* urethra. (Modified from: Muro et al. 2021b)

**Fig. 4:**
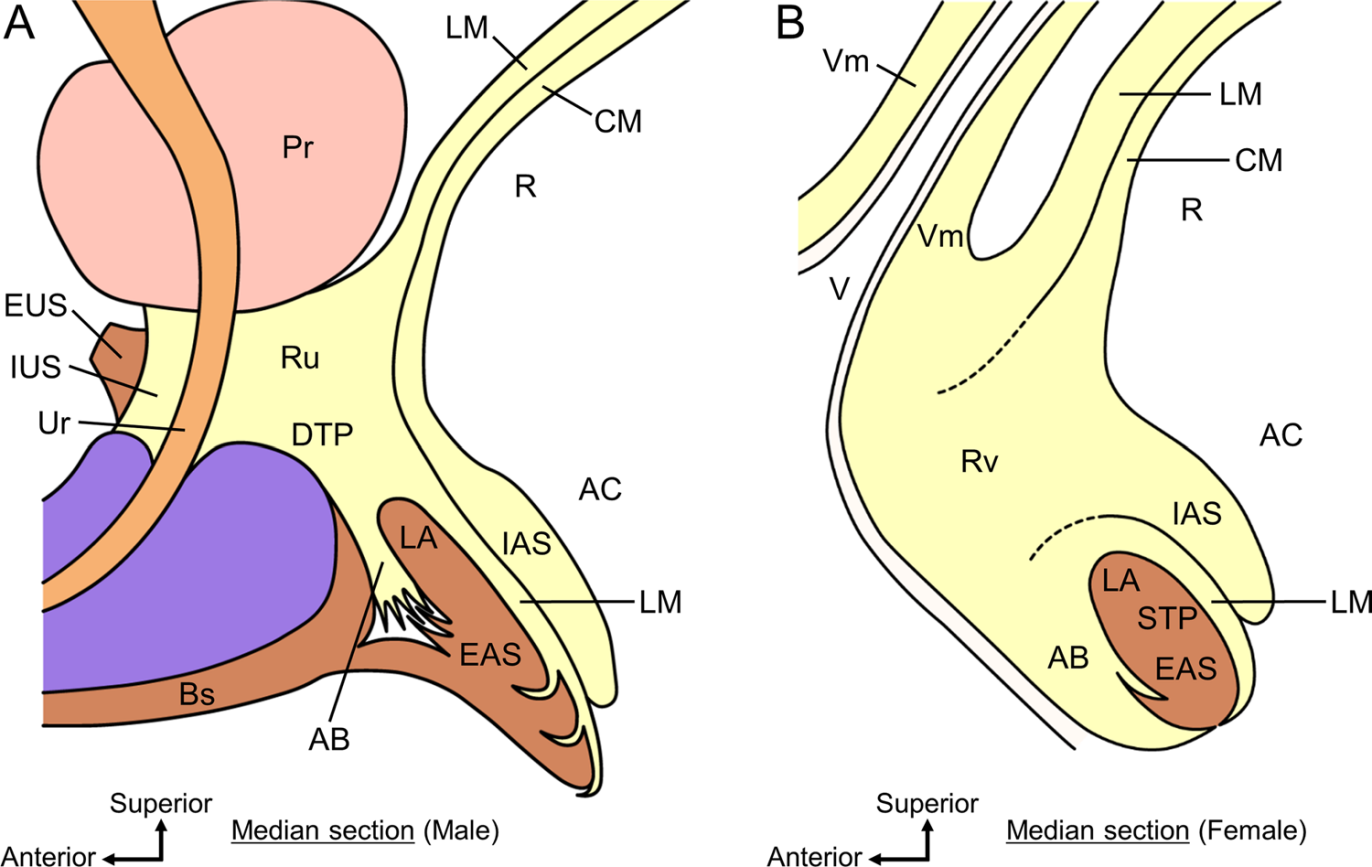
Comparison of smooth muscle in the region anterior to the rectum and anal canal in men and women. **A** Median section of the region anterior to the rectum and anal canal in men. The Ru is continuous posteriorly with the LM and anteriorly with the IUS, that is, it connects the rectum to the urethra. The AB in men extends anteroinferior from the LM and is located between the EAS and Bs, covering the anterosuperior surface of the EAS. The smooth muscle tissue between the Ru and AB corresponds to the median part of the DTP. **B** Median section of the region anterior to the rectum and anal canal in women. The Rv extends from the LM and CM of the rectum and anal canal, including the IAS, intermingles with the Vm of the lower posterior vaginal wall, and spreads subcutaneously in the vaginal vestibule and perineum. It is considered to be a structure homologous to Ru in men. The AB in women extends anteroinferior from the LM and diffuses anterior to the EAS and subcutaneously in the perineum, completely covering the anterior surface of the EAS. *AB* anterior bundle of LM, *AC* anal canal, *ACL* anococcygeal ligament, *Bs* bulbospongiosus, *CM* circular muscle, *DTP* deep transverse perineal muscle, *EAS* external anal sphincter, *EUS* external urethral sphincter, *HL* hiatal ligament, *IAS* internal anal sphincter, *IUS* internal urethral sphincter, *LA* levator ani, *LM* longitudinal muscle, *Pr* prostate, *R* rectum, *Ru* rectourethralis, *Rv* rectovaginalis, *STP* superficial transverse perineal muscle, *SV* seminal vesicle, *Ur* urethra, *V* vagina, *Vm* vaginal muscularis (muscle layer of the vagina) (Modified from: Muro et al. 2018, 2019)

The wedge-shaped structure called the “rectourethralis” is located between the rectum and urethra in men (Figs. 3 and 4) (Brooks et al. 2002; Matsubara et al. 2003; Muro et al. 2018; Nakajima et al. 2017; Nyangoh Timoh et al. 2020; Okada et al. 2019; Porzionato et al. 2005; Rosse et al. 1997; Soga et al. 2008; Standring and Gray 2015; Uchimoto et al. 2007; Zhai et al. 2011). It is composed of smooth muscle fibers that extend from the longitudinal muscle (LM) of the rectum to the smooth muscle of the urethra. It has sparsely scattered smooth muscle fibers that run dorsoventrally. It connects the urethra to the rectum. Given that it links the rectal wall to the membranous urethra, the rectourethralis is thought to assist in stabilizing the urethra and to be a factor in the anorectal flexure (Brooks et al. 2002; Roux 1881; Soga et al. 2008; Zhai et al. 2011).

In women, the space between the anorectal canal and vagina is occupied by smooth muscle tissues (Fig. 4B) (Aigner et al. 2004; Kinugasa et al. 2013; Muro et al. 2019; Oh and Kark 1973). This smooth muscle extends from the longitudinal and circular muscles of the rectum and anal canal, including the internal anal sphincter (IAS), intermingles with the smooth muscle layer of the lower posterior vaginal wall and spreads subcutaneously in the vaginal vestibule and perineum (Muro et al. 2019). Oh and Kark called this smooth muscle “rectovaginalis” (Oh and Kark 1973). The rectovaginalis seems to contribute to the stabilization of the lower part of the vaginal posterior wall and is likely one of the key structures for preventing vaginal prolapse (Muro et al. 2019). The rectourethralis in men and the rectovaginalis in women are considered homologous structures based on location, morphology, and tissue composition (Fig. 4).

#### Deep transverse perineal muscle

**Fig. 5:**
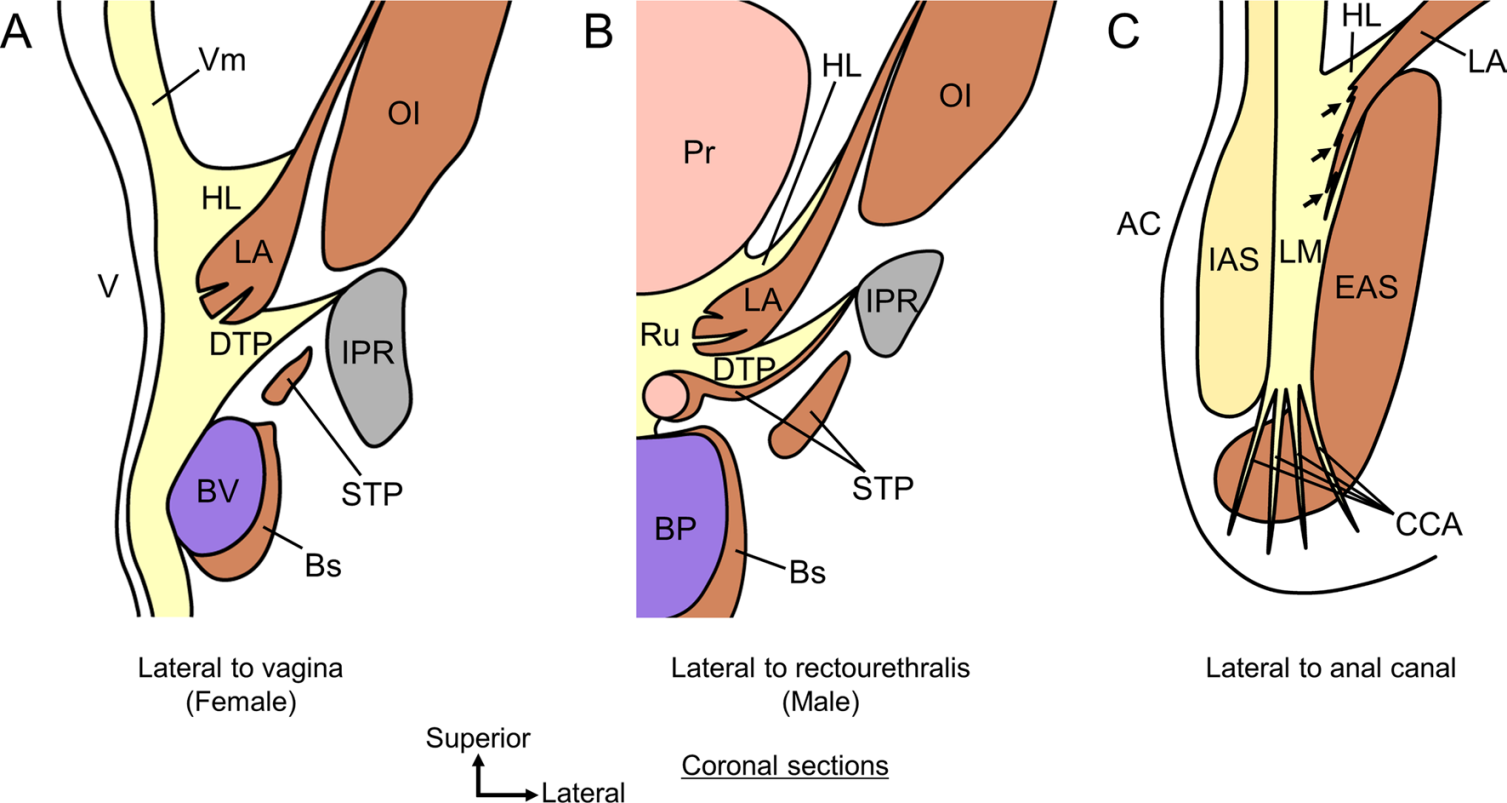
Interface between levator ani and pelvic viscera. LA is sandwiched superomedially and inferolaterally by smooth muscle extending from the visceral wall. **A** Coronal section through the vagina. The LA is sandwiched between the smooth muscle of the HL and DTP, which is formed by smooth muscle fibers extending superiorly and inferiorly from the vaginal wall to the LA. The DTP is a smooth muscle plate-like structure in the deep perineal pouch inferior to the LA. **B** Coronal section through the rectourethralis. The LA is sandwiched between the smooth muscle of the HL and DTP, which is formed by smooth muscle fibers extending from Ru superiorly and inferiorly to the LA. The DTP is a smooth muscle plate-like structure in the deep perineal pouch inferior to the LA. **C** Coronal section through the anal canal. The skeletal muscle fibers of the LA directly attach to the smooth muscle of the rectal wall and anal canal (arrows). The LA is sandwiched between the smooth muscle of the HL and slight smooth muscle fibers entering between the LM and EAS, which are formed by smooth muscle fibers extending from the LM superomedially and inferolaterally to the LA. Several smooth muscle fibers of the LM penetrated the EAS inferiorly and mainly spread subcutaneously around the anus; these fibers are called “corrugator cutis ani”. Arrows, direct attachment of skeletal muscle and smooth muscle; *AC* anal canal, *Bs* bulbospongiosus, *BP* bulb of the penis, *BV* bulb of the vestibule, *CCA* corrugator cutis ani, *DTP* deep transverse perineal muscle, *EAS* external anal sphincter, *HL* hiatal ligament, *IAS* internal anal sphincter, *IPR* ischiopubic ramus, *LA* levator ani, *LM* longitudinal muscle, *OI* obturator internus, *Pr* prostate, *Ru* rectourethralis, *STP* superficial transverse perineal muscle, *V* vagina, *Vm* vaginal muscularis (muscle layer of the vagina). (Modified from: Muro et al. 2018)

The plate-like structure called the “deep transverse perineal muscle” (DTP) is located between the rectum and urethra caudal to the rectourethralis/rectovaginalis (Figs. 3, 4, and 5). It crosses the midline, spreads bilaterally, and reaches the ischiopubic rami (Muro et al. 2018, 2021a, 2021b; Nyangoh Timoh et al. 2020; Wu et al. 2017, 2018; Zhai et al. 2011). It consists of smooth muscle fibers that extend from the LM of the rectum and anal canal. The smooth muscle fibers are transversely oriented and densely packed. The DTP is located in the deep perineal pouch and surrounded superiorly by the LA and inferiorly by the perineal membrane.

The DTP muscle, also known as the musculus transversus perinei profundus, is generally described to be a skeletal muscle that arises from the ischiopubic rami and runs medially (Clemente 1985). Previous studies on DTP have shown conflicting findings regarding the presence and histological composition of skeletal muscle or smooth muscle (Arakawa et al. 2010; Courtney 1950; Kokoua et al. 1993; Murakami et al. 2002; Nakajima et al. 2007). However, in more recent publications, DTP has frequently been used to refer to smooth muscle plate-like structures in the deep perineal pouch (Figs. 3 and 5) (Zhai et al. 2011; Wu et al. 2017, 2018; Muro et al. 2018, 2021b; Nyangoh Timoh et al. 2020). The novel interpretation of DTP, which consists of smooth muscle that extends from the midline to the ischiopubic rami and is situated anteroinferior to the LA, is given by the morphology of the series of smooth muscle structures: the DTP muscle. The anatomical features of the DTP, such as its continuity with the LM of the rectum and its crossing of the midline, may be important when considering the pelvic floor support mechanism (Muro et al. 2019, 2021b). The DTP appears to be important as a structure that connects various skeletal muscles together and as a key component of the dynamic coordination between the skeletal and smooth muscles discussed below.

#### Anterior bundle of longitudinal muscle

The columnar structure, which is described as the “anterior bundle of the LM” (AB), is located in the median region between EAS and Bs in men (Fig. 4A) (Aigner et al. 2004; Muro et al. 2018, 2021b; Nakajima et al. 2017; Nyangoh Timoh et al. 2020; Smith 1908; Zhai et al. 2011). It covered the anterosuperior surface of the EAS. The AB consists of smooth muscle fibers that extend from the LM of the rectum and anal canal. The smooth muscle fibers run vertically (craniocaudal direction) and are densely packed. The skeletal muscle fibers of the perineal muscles (Bs, STP, and EAS) surround AB. Although there are multiple reports on AB, it is not well known. AB is a small element, and its functional significance is unclear. However, it may support the central part of the perineum by lifting between the EAS and Bs. It is difficult to visualize AB using CT or MRI because it is a very small element. The success of Nakajima et al. in delineating AB using transanal ultrasonography in both living bodies and cadavers has important implications for both clinical diagnosis and anatomical analysis (Nakajima et al. 2017).

AB is mostly reported in men; however, its corresponding structures are also present in women (Fig. 4B) (Muro et al. 2019). The AB in women, as well as in men, consists of smooth muscle extending anteriorly from the LM of the rectum and anal canal and covers the anterior surface of the EAS. However, in women, because Bs are not located in the median anterior to AB, they are not sandwiched between Bs and EAS as in men, but rather extend diffusely anterior to the EAS and subcutaneously in the perineum. Thus, the AB in women is wider than that in men, completely covering the anterior surface of the EAS and encompassing the EAS along with the LM of the anal canal, which is internal to the EAS. Although we cannot assert its functional importance from morphology alone, we can speculate from these morphological features of the AB that this is likely to be important as a supporting element for the anal canal and pelvic viscera.

#### Hiatal ligament

The amorphous tissue called the “hiatal ligament,” is located between the pelvic viscera (urethra, vagina, rectum, and anal canal) and LA; in other words, it fills the gap (hiatus) between them (Arakawa et al. 2010; Murakami et al. 2002; Muro et al. 2014, 2018, 2021b; Nyangoh Timoh et al. 2018; Shafik 1975, 1979, 1999; Tsukada et al. 2016). It is composed of smooth muscle fibers that extend from the vaginal wall, rectourethralis, and LM of the rectum. Thus, for example, there is no clear boundary between the hiatal ligament and the LM of the rectum (Tsukada et al. 2016). The smooth muscle fibers of the hiatal ligament run in the superolateral direction and are sparsely scattered. The hiatal ligament contacts the internal surface of the LA and is recognized as an intermediary with the pelvic viscera; thus, is considered to complement or facilitate the attachment of the LA to the pelvic viscera (Shafik 1975, 1979, 1999).

The hiatal ligament is thickened and distinct posteriorly to the rectum, and the thickened part is also called the “recto-coccygeus” (Courtney 1948, 1949; Kinugasa et al. 2011, 2012; Rosse et al. 1997; Standring and Gray 2015). It extends from the LM of the rectum, covers the internal surface of the LA, and reaches the ventral side of the coccyx.

#### Corrugator cutis ani and anococcygeal ligament

Several fibers or septa are located between the muscle bundles of the subcutaneous part of the EAS (Fig. 5C) (Lawson 1974; Muro et al. 2014, 2020; Shafik 1976; Tsukada et al. 2016). They consist of smooth muscle fibers directly extending from the LM of the anal canal; in other words, the fibers are the inferior extending structure of the LM. They penetrated the subcutaneous part of the EAS and spread subcutaneously around the anus. Some older textbooks and articles refer to this as “corrugator cutis ani” (Clemente 1985; Shafik 1976). The corrugator cutis ani is considered to lift and evert the EAS and anal orifice during defecation (Lunniss and Phillips 1992; Shafik 1976).

**Fig. 6:**
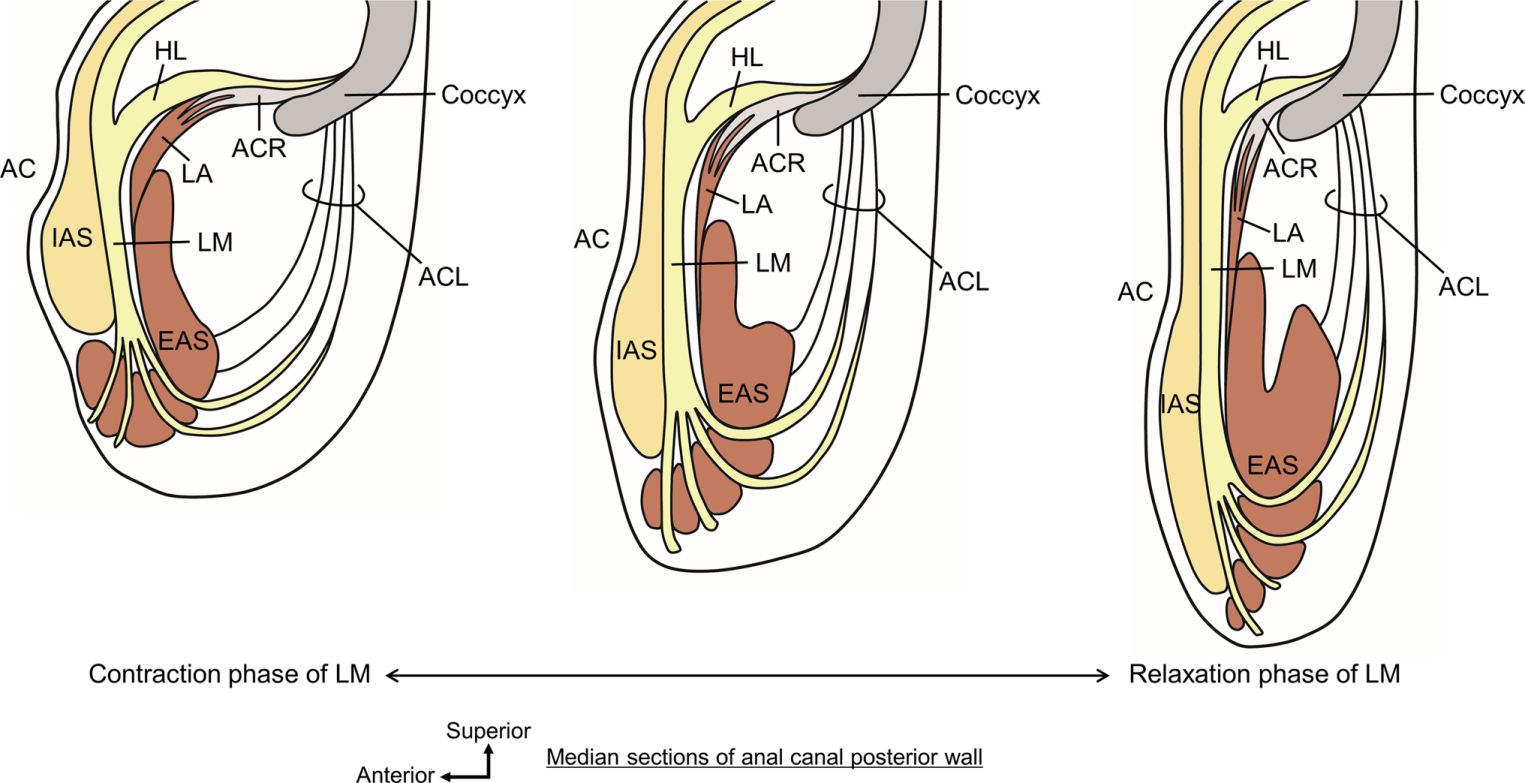
The suspension structure of the external anal sphincter posterior to the anal canal by longitudinal muscle. These schemata show the sagittal sections of the region posterior to the anal canal. Some smooth muscle fibers extending from the LM that penetrate the EAS connect to the ACL, which consists of collagenous and elastic fibers that lead to the coccyx. This suspension structure suggests that contraction and relaxation of the LM change the shape of the EAS via the ACL. In the contraction phase of the LM, it contracts and elevates the subcutaneous part of the EAS; thus, the subcutaneous part of the EAS bends anteriorly and is located inferior to the IAS. The ACL is tensionless; therefore, it appears as a gentle curve from the muscles of the anal canal to the coccyx. In the relaxation phase, the LM relaxes and does not elevate the subcutaneous part of EAS. This increases the tension of the ACL, and the subcutaneous part of the EAS is slung posteriorly by the ACL, which makes the EAS twofold. The ACL appeared as a relatively straight line from the EAS to the coccyx. *AC* anal canal, *ACL* anococcygeal ligament, *ACR* anococcygeal raphe, *EAS* external anal sphincter, *HL* hiatal ligament, *IAS* internal anal sphincter, *LA* levator ani, *LM* longitudinal muscle. (Modified from: Muro et al. 2014)

Smooth muscle fibers penetrating the subcutaneous part of the EAS are observed around the anal canal; however, the posterior part of the anal canal is unique. Posterior to the anal canal, some smooth muscle fibers that penetrate the subcutaneous part of the EAS connect to collagenous and elastic fibers, leading to the dorsal surface of the coccyx (Fig. 6) (Ayoub 1979a; Muro et al. 2014; Toldt 1903). These fibers are called “anococcygeal ligaments” because they connect the anal canal to the coccyx. Using the anococcygeal ligament as a core, the muscle bundles of EAS converge posteriorly and extended superoposteriorly to attach to the dorsal surface of the coccyx (Suriyut et al. 2020). The inferior extending fibers of the LM that penetrate the EAS and the anococcygeal ligament form the suspension structure of the EAS posterior to the anal canal by LM. Muro et al. (2014) reported two types of variations in the shape of EAS in the posterior wall of the anal canal, which they interpreted as different phases of the same structure (Fig. 6) (Muro et al. 2014). Differences in the shape of the EAS and pathway of the anococcygeal ligament were observed correspondingly and simultaneously, suggesting that the contraction and relaxation of the LM change the shape of the EAS via the anococcygeal ligament. This suggests a dynamic interaction between the smooth and skeletal muscles, whereby the smooth muscle changes the shape of the skeletal muscle.

### Origin of smooth muscles

Where did the smooth muscle that occupies the central region of the pelvic floor and perineum come from? There are two hypotheses regarding its origin: one holds that the smooth muscle originates from the fascia of the LA, and the other holds that it originates from the smooth muscle of the visceral wall. The former considers that the covering fascia of the LA, including the smooth muscles, has extended to the central region of the pelvic floor and perineum, based on morphological findings that smooth muscle tissue covers the surface of the LA (Arakawa et al. 2004, 2010; Murakami et al. 2002; Uchimoto et al. 2007). The latter considers that the smooth muscle of the pelvic viscera extends into the surroundings and central region of the pelvic floor and perineum, based on the morphological findings that smooth muscle tissue is continuous with the smooth muscle that consists of the walls of the rectum and vagina (Kato et al. 2020; Muro et al. 2014, 2018, 2019, 2021b; Nakajima et al. 2017; Tsukada et al. 2016). Because the smooth muscle of the pelvic outlet is continuous with the walls of the pelvic viscera and, as described below, is also closely associated with the LA, it is difficult to determine the origin of the smooth muscle by observing its structure. However, embryological studies and macroscopic examination of the anal canal muscles support the latter hypothesis.

Studies involving human embryos and fetuses have demonstrated that the smooth muscle of the LM of the rectum extends to surrounding structures at 8–9 weeks of embryonic development (Fritsch et al. 2007; Sebe et al. 2005). This suggests that a part of the smooth muscle in the central region of the pelvic floor and perineum is formed as an extension of the LM. Additionally, a detailed anatomical study using adult cadavers clarified that, in the anterior wall of the rectum and anal canal, the muscle bundles of the IAS and the LM showed a convergent course toward the anterior anal wall and extended anteriorly (Muro et al. 2019). Even if the smooth muscle derived from the fascia of the LA was attached to the rectal wall, it did not significantly alter the running of the muscle bundles of the rectum. The characteristic convergent course of the muscle bundles is associated with the anterior extension of the IAS and LM, suggesting that the smooth muscle extends from the wall of the rectum and anal canal to the surrounding area. Furthermore, in the area where this characteristic convergence of muscle bundles was observed, a characteristic structure was found in embryological analysis (Long et al. 2020; Sasaki et al. 2004). When the cloaca is divided by the urorectal septum, a canal is momentarily established between the urogenital sinus and distal portion of the hindgut, according to research on mouse or rat embryos. This temporary communication structure between the urogenital and digestive tract regions may allow the smooth muscle from the rectal wall to extend anteriorly.

In contrast, it is also true that, on observation of histological sections (especially horizontal sections), smooth muscle tissue may appear as if it were attributed to the LA and poorly related to the smooth muscle of the visceral wall. Underlying such observations is the coexistence of dense and sparse areas within the continuous extension of the smooth muscle tissue (Muro et al. 2020). As discussed below, the smooth muscle closely associated with the LA is relatively sparse, whereas the smooth muscle that composes the visceral wall is relatively dense. Failure to recognize the coexistence of dense and sparse areas within the smooth muscle leads to the fallacy of thinking of each as a separate structure.

These findings suggest that the smooth muscle that occupies the central region of the pelvic floor and perineum does not originate from the fascia of the LA but from the smooth muscle of the visceral wall. Additionally, these smooth muscle tissues are also found in fetal specimens, indicating that this is not an acquired structure like granulation tissue, but a structure that is formed during the developmental process (Fritsch et al. 2007; Nyangoh Timoh et al. 2018, 2020, 2021; Sebe et al. 2005). To understand the structure of the pelvic floor and perineum, the extended nature of smooth muscles must be recognized.

### Dense and sparse areas of smooth muscle tissues

**Fig. 7:**
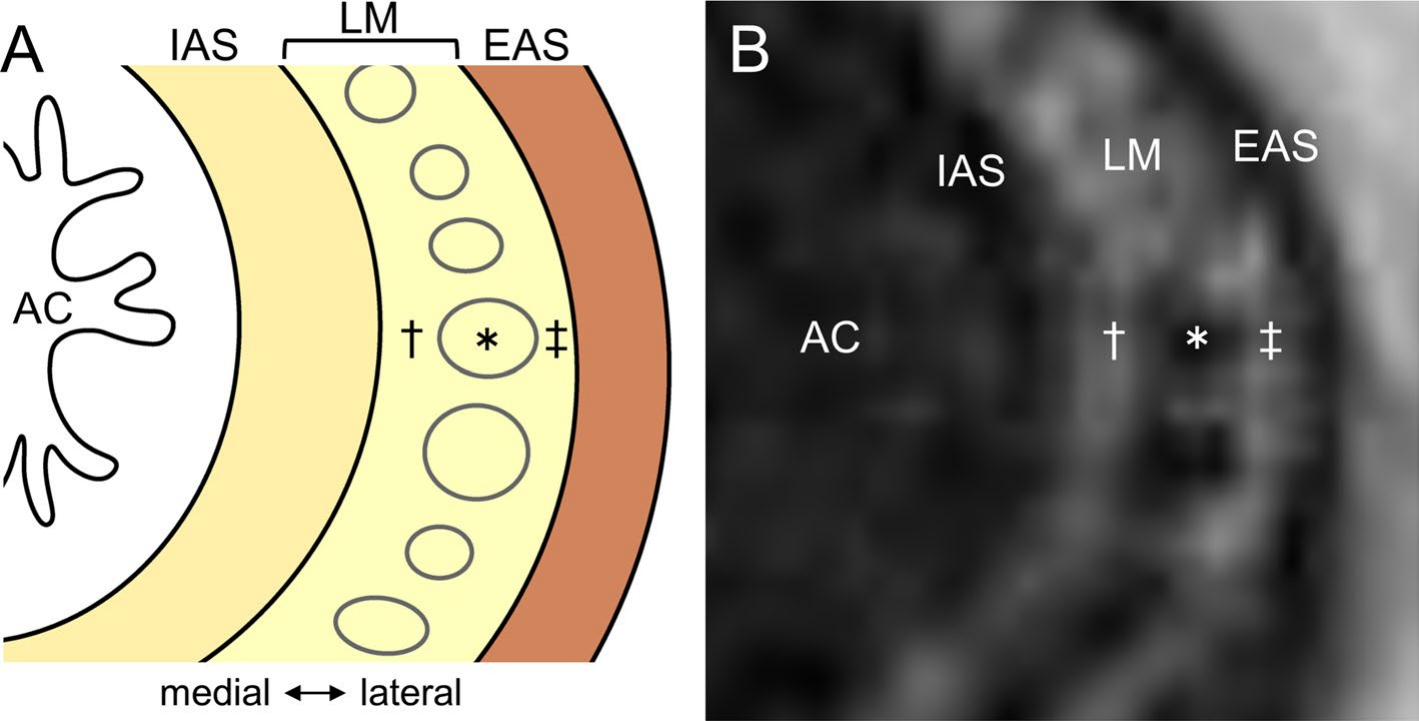
Coexistence of dense and sparse areas of smooth muscle in the longitudinal muscle layer of the anal canal. **A** Schema of the transverse (axial) section of the anal canal. The LM is the muscle layer between the IAS and EAS. There are dense and sparse areas of smooth muscle in LM. **B** Magnetic resonance image of the transverse (axial) section of the anal canal. The dense and sparse areas of the smooth muscle in the LM are depicted by MRI. The dense areas are depicted as low-intensity areas, and the sparse areas are depicted as high-intensity areas. *AC* anal canal, *EAS* external anal sphincter, *IAS* internal anal sphincter, *LM* longitudinal muscle, *MRI* magnetic resonance imaging, asterisk (*), dense area; obelisk (†) and double obelisk (‡), sparse area. (Modified from: Muro et al. 2020)

The fiber orientations and densities of the series of smooth muscle structures in the pelvic outlet varied according to location. Fiber orientation is thought to be reflected in the direction of smooth muscle contraction, and fiber density in the intensity of smooth muscle contraction. In particular, focusing on the fiber density of the smooth muscles is a new perspective. For example, in the smooth muscle elements described above, the DTP and AB are relatively dense, whereas the rectourethralis and hiatal ligament are relatively sparse (Muro et al. 2021b) (Table 2). In the rectovaginalis of women, the median portion is relatively dense, whereas the lateral portion is relatively sparse, with blood vessels and nerves passing through (Muro et al. 2019). Therefore, the smooth muscle of the pelvic outlet is not uniform, although it extends continuously, with dense and sparse areas within the continuous expansion. Moreover, not only in these extended structures but also in the smooth muscle, which consists of the wall of the viscera, dense and sparse areas coexist. It is located in the LM layer of the anal canal (Fig. 7) (Muro et al. 2020, 2021b). The dense area in the LM of the anal canal is directly continuous with the muscle bundles of the LM of the rectum and can be interpreted as gut-specific longitudinal muscle. The sparse area is directly attached to the skeletal muscle fibers of the LA and can be interpreted as smooth muscle tissue responsible for adhesion to the levator ani (the direct attachment with skeletal muscle, as described below). This difference in density seems to be optimized for the function of each part of the smooth muscle; dense smooth muscle fibers are optimized for shortening the anal canal by forming dense bundles, and sparse smooth muscle fibers for fixation with skeletal muscles by forming flexible shapes. These dense and sparse areas of smooth muscle in the LM of the anal canal were also depicted by MRI (Muro et al. 2020). Differences in density are reflected in the differences in MRI intensity.

Additionally, the smooth muscle structure extending in the pelvic outlet region may be classified between “connective tissue” and “muscle tissue” of the conventional histological classification. For example, the inner circular and outer longitudinal muscles of the digestive tract have smooth muscle fibers regularly arranged in a certain bundle. However, the smooth muscle structure extending in the pelvic outlet region has fibers spread vaguely and does not form bundles, although their direction is recognizable. It has dense and sparse areas, and it shows a spread that occupies other structures, such as skeletal muscles and viscera. Such a property is interpreted as “connective tissue-likeness.” However, the main component of this structure is the smooth muscle fibers. This indicates that they are capable of contracting. This point is interpreted as “muscle tissue-likeness.” From these points of view, the smooth muscle structure extending in the pelvic outlet region may be classified as an intermediate tissue between “connective tissue” and “muscle tissue.” In recent years, it has been proposed to analyze connective tissues, such as fascia and ligaments, based on differences in the regularity and density of fibers (Schleip et al. 2012). It may be possible to adapt this perspective for the analysis of smooth muscles.

## Interface between levator ani and pelvic viscera

When considering the pelvic floor support mechanism, it is important to consider how the LA and pelvic viscera are in contact with each other. At the interface between the LA and pelvic viscera, the following four characteristic structures are formed by the smooth muscles. Such smooth muscle structures seem to intervene between the LA and the pelvic viscera, transferring the lifting power of the LA to the pelvic viscera. These are thought to be unique to the pelvic region, where the smooth muscles of the viscera and skeletal muscles of the pelvic wall are in close proximity.

### Direct attachment of skeletal and smooth muscles

As aforementioned, the LA contains muscle bundles that attach to the pelvic viscus, specifically the rectal wall (Kearney et al. 2004; Lawson 1974; Smith 1923). Where the fibers of LA attach to the rectal wall, direct attachment of smooth and skeletal muscles can be observed: the smooth muscle fibers of LM attach directly to the skeletal muscle fibers of LA (indicated by the arrows in Fig. 5C) (Muro et al. 2020; Tsukada et al. 2016). Such a direct attachment is prominent on the anterolateral wall of the rectum, perpendicular to the muscle bundles of the LA. The discovery of direct attachment overturned the classical concept of the “conjoined longitudinal muscle” of the anal canal. Several previous studies have reported that fibers of the LM of the rectum blend with fibers of the LA, thereby forming a conjoined (Gorsch 1960; Lawson 1974; Lunniss and Phillips 1992), combined (Courtney 1950), or conjoint (Gray et al. 2008) LM layer between the IAS and EAS. However, recently it has been clarified that LM and LA do not mix, but that different tissues, smooth and skeletal muscles directly attach (Muro et al. 2020; Tsukada et al. 2016).

### Skeletal muscle sandwiched by smooth muscle

The LA is sandwiched superiorly and inferiorly (or medially and laterally) by smooth muscles extending from the visceral wall. This sandwiching of skeletal muscle by the smooth muscle is found in locations where the aforementioned direct attachments are few or absent: the wall of the vagina, lateral and posterior walls of the rectum, and more interestingly, lateral to the rectourethralis (Fig. 5) (Kato et al. 2020; Muro et al. 2018). The LA faces smooth muscle tissue on its superomedial and inferolateral surfaces, and the smooth muscle that extends over the superomedial surface of the LA corresponds to the hiatal ligament. The smooth muscle that extends inferior to the LA develops in the deep perineal pouch and corresponds to the DTP described above. For example, several smooth muscle fibers extending from the vaginal wall extended superiorly and inferiorly to the LA (Fig. 5A). Consequently, the LA is sandwiched between smooth muscle fibers both superiorly and inferiorly.

### Smooth muscle insertion into skeletal muscle

A few fibers of smooth muscle, extending from the visceral wall, are inserted between the muscle bundles of the LA. Such insertion into the skeletal muscle by the smooth muscle is found around the vagina, rectum, anal canal, and lateral to the rectourethralis (Kato et al. 2020; Muro et al. 2018). This inserted smooth muscle forms a strongly interlocked structure between the pelvic viscera and LA and seems to act as an intervening structure that transmits the force of the skeletal muscles of the LA to the pelvic viscera.

### Complementary relationship between direct attachment and sandwiching (hiatal ligament)

Direct attachment is observed predominantly in the anterolateral wall of the rectum, where the fibers of the LA attach to the rectal wall, whereas the sandwiching of the skeletal muscle by smooth muscle, including the hiatal ligament, is observed predominantly in the lateral and posterior walls of the rectum and around the vagina. Based on these findings, it appears as if the sandwiching compensates for the adhesion of the LA to the pelvic viscera where the direct attachment is scarce or absent. In other words, direct attachment and sandwiching (hiatal ligament) have a complementary relationship. The complementary relationship between the direct attachment and hiatal ligament around the rectum has been reported by Tsukada et al. (2016). The width of the direct attachment and thickness of the hiatal ligament differed in different portions of the rectum. In the anterolateral portion of the rectum, the direct attachment is wide and the hiatal ligament is thin. In contrast, in the posterior portion of the rectum, the direct attachment is narrower, and the hiatal ligament is thicker.

## Complementary arrangement of skeletal and smooth muscles

The smooth muscles of the pelvic outlet extend to fill the spaces between two skeletal muscles, resulting in the complementary arrangement of skeletal and smooth muscles in various regions of the pelvic outlet, as in the region anterior to the anal canal, as reported by Nakajima et al. (2017). Such a complementary arrangement seems to create strong adhesion between the smooth and skeletal muscles and the possibility of interlocking them.

In the region anterior to the anal canal in men, the IAS and LM (smooth muscle) are located on the luminal side, and the EAS (skeletal muscle) is located anterior to them. AB (smooth muscle) is located anterior to the EAS, and Bs (skeletal muscle) is located further anterior to it (Fig. 4A) (Nakajima et al. 2017). By complementarily arising smooth muscle and skeletal muscle in the anteroposterior direction in this manner, the region anterior to the anal canal is occupied with muscles, and the muscles are in contact with each other. This arrangement of muscles may contribute to the stabilization of the region anterior to the anal canal, that is, the center of the perineum. Although Bs is not observed in the region anterior to the anal canal in women, the sequence is similar to that of men from the luminal side of the anal canal anteriorly: IAS, LM, EAS, and AB (Fig. 4B) (Muro et al. 2019). The smooth muscles LM and AB surround the EAS anteriorly and posteriorly. Although we cannot affirm its functional importance only based on morphology, this complementary arrangement of skeletal and smooth muscles imply that this is likely to be important as a supporting element for the anal canal.

The corrugator cutis ani (Fig. 5C) and anococcygeal ligament (Fig. 6) in the lower part of the anal canal are also complementary arrangements at a smaller level (Muro et al. 2014). Smooth muscle fibers extending inferiorly from the LM penetrate the EAS, resulting in a complementary arrangement of smooth muscle fibers and skeletal muscle bundles of the EAS. With this type of structure, the LM movement is transmitted to the EAS, enabling linked movement. Sandwiching of skeletal muscle by smooth muscle, as described above, can be understood as a complementary arrangement (Kato et al. 2020; Muro et al. 2018, 2021b). If the STP is also considered together, the layered structure is composed of the hiatal ligament, LA, DTP, and STP from superior to inferior, with smooth and skeletal muscle complementarily represented (Fig. 5A and B). In the complementary arrangement, the smooth and skeletal muscles are sandwiched between each other. Owing to this muscle arrangement, the LA is tightly bound to the smooth muscle. Then, the movement of the LA is transmitted to the pelvic viscera via smooth muscles such as the hiatal ligament and DTP. Additionally, contraction or relaxation of a series of smooth muscle structures in the center of the pelvic outlet is transmitted to the LA.

Secretory glands are sometimes interspersed within such complementary arrangements. Muro et al. (2018, 2021a) reported that Cowper’s gland (bulbourethral gland) in men is surrounded by skeletal muscle on the lateral side and smooth muscle on the posterosuperior and medial sides (Muro et al. 2018, 2021a). This skeletal muscle is a part of a continuous structure composed of EUS, STP, EAS, and LA muscle bundles, and the smooth muscle is the DTP. The anatomy of the muscles surrounding the Cowper’s gland has been a subject of debate. Some state that DTP surrounds the Cowper’s gland, while others state that EUS surrounds it (Clemente 1985; Cunningham and Romanes 1981; Standring and Gray 2015; Williams 1995). Both of these conventional statements are partially correct. The Cowper’s gland is surrounded by both skeletal and smooth muscles, and the complementary arrangement of skeletal and smooth muscles unique to the pelvic outlet is formed around the Cowper’s gland. These findings suggest that skeletal and smooth muscles work together to carry out the secretion and emission of Cowper’s gland. Interestingly, skeletal and smooth muscles work in cooperation for glandular function.

## Dynamic coordination of skeletal and smooth muscles

Conventionally, the mechanism of pelvic floor support has been discussed primarily through the skeletal muscles and ligaments (DeLancey 1993, 1994; Norton 1993; Petros and Ulmsten 1990; Petros and Woodman 2008; Zacharin 1963). However, even those previously described as ligaments have been reported to contain many smooth muscle cells (uterosacral ligaments) (Gabriel et al. 2005). Moreover, as aforementioned, the smooth muscle is extensively spread throughout the center region of the pelvic outlet (Kato et al. 2020; Muro et al. 2014, 2018, 2019, 2020, 2021a, 2021b; Nakajima et al. 2017; Tsukada et al. 2016). What has traditionally been described as ligaments may be part of these smooth muscle elements. Based on the novel anatomical understanding of the skeletal and smooth muscles of the pelvic outlet, the contractile capacity of the smooth muscle and its close relationship with skeletal muscle cannot be ignored. Undoubtedly, skeletal muscle is the main player in pelvic floor support; however, we propose smooth muscle as the fundamental carrier of pelvic floor support.

In general, smooth muscle contraction mechanisms can be envisioned as follows: 1) contraction under the influence of neural signals, and 2) contraction in the absence of neural signals (Bayliss effect). It has been reported that smooth muscle structures extending to the center of the pelvic outlet are innervated by autonomic nerves arising from the pelvic plexus (inferior hypogastric plexus) (He et al. 2022; Nyangoh Timoh et al. 2020). Given that these smooth muscles extend continuously from the LM of the rectum, it would be expected that the nerve innervating the smooth muscle structures would also be closely associated with the rectal branch of the pelvic plexus. At present, however, the details of the innervating nerve remain unclear. Further analysis of this innervation is expected in the future. The Bayliss effect is an autocontractile response of smooth muscle, which is mainly referred to in the smooth muscle of blood vessels (Bayliss 1902; Ji et al. 2002; Nelson 1998). It has long been known physiologically that smooth muscle responds with contraction to sudden stretching without any influence from external nerves or humoral factors. Given the Bayliss effect on smooth muscle, the spread of smooth muscle in the pelvic outlet appears to be of great functional significance. The pelvic floor receives sudden pressure (sudden stretching) owing to abdominal pressure and other factors, similar to the way the vessel wall receives blood pressure. Therefore, it is probably a functionally significant characteristic of this region to react with contraction instantaneously to resist abdominal pressure without requiring nerve signals, and it is a reasonable structure for this function.

**Fig. 8:**
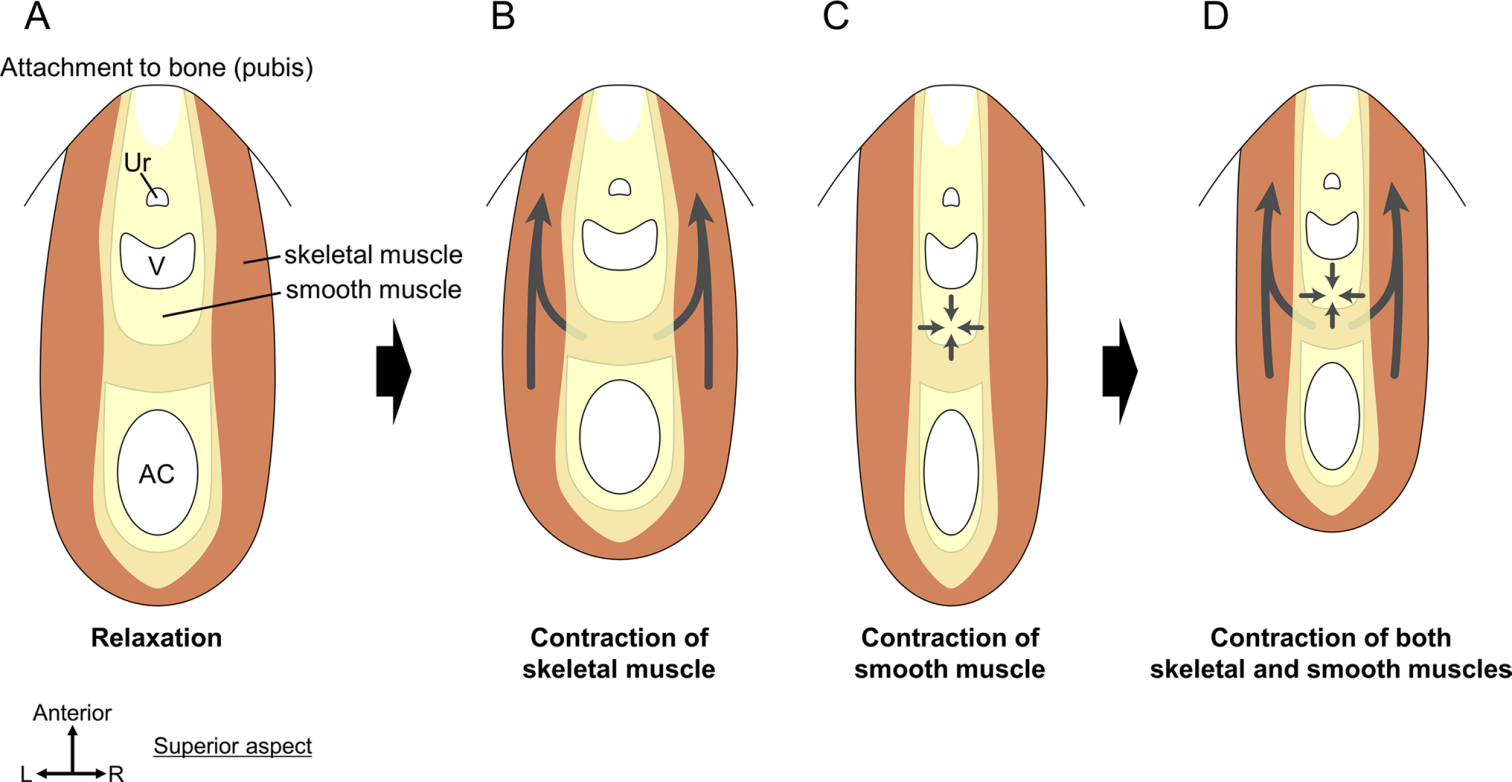
Dynamic coordination of skeletal and smooth muscles in the pelvic outlet. A hypothetical model of the interaction between skeletal and smooth muscles in the pelvic outlet. Skeletal muscle is shown in brown and smooth muscle in yellow, in this view of the pelvic outlet from superior. These two muscle types are in close contact with each other. When skeletal muscle contracts, the shape of the smooth muscle change, and the direction, angle, and density of the smooth muscle fibers change. When the smooth muscle contracts, the direction, angle, and length of skeletal muscle bundles change. The skeletal and smooth muscles will contract and relax while regulating each other’s direction and force. *AC* anal canal, *Ur* urethra, *V* vagina

Furthermore, the close relationship between smooth and skeletal muscles is noteworthy. These two muscle types are in close contact with each other, which suggests that the skeletal muscles change shape as the central smooth muscles contract or relax. In other words, the direction, angle, and length of the skeletal muscle bundles in the pelvic outlet can change as a result of the contraction or relaxation of smooth muscles in the central region (Fig. 8). A hypothetical model is presented in Fig. 8 which shows a view of the pelvic outlet from superior. The urethra, vagina, and anal canal are surrounded by skeletal muscle (equivalent to the LA), with smooth muscle filling in between (Fig. 8A). When the skeletal muscle contracts, it would not only pull the viscera anteriorly, but also change the shape of the smooth muscle (Fig. 8B). On the other hand, contraction of the smooth muscle would pull the skeletal muscle toward the center of the pelvic outlet and change the orientation and angle of the skeletal muscle (Fig. 8C). Thus, the skeletal and smooth muscles would contract and relax while regulating each other's direction and force (Fig. 8D). By coordinating the extended smooth muscle with a continuous skeletal muscle sheet, the pelvic floor most likely produces functional dynamism. According to Fritsch et al. (2004), pelvic floor support is multi-structural (Fritsch et al. 2004). A novel anatomical understanding of the pelvic outlet muscles suggests muscular multi-structure, including different types of muscles, that is, skeletal and smooth, which are responsible for the pelvic floor mechanism. This is the dynamic coordination between the skeletal and smooth muscles.

## Conclusion

The anatomy of the pelvic floor has previously been described in terms of the skeletal muscles and ligaments. Recently, however, the detailed anatomy of smooth muscles in the pelvic floor and perineum has been examined, presenting a novel concept of pelvic floor structure and function: the dynamic coordination between the skeletal and smooth muscles. If this new anatomical concept enables the redefinition of the pathophysiology of pelvic organ prolapse, urinary incontinence, and fecal incontinence, it may lead to the creation of better preventive and early diagnostic indicators.

## Data Availability

All the relevant data used in this study can be accessed upon reasonable request from the corresponding author.
